# UNDERSTANDING THE INTERNATIONAL CONSENSUS FOR ACUTE PANCREATITIS: CLASSIFICATION OF ATLANTA 2012

**DOI:** 10.1590/0102-6720201600030018

**Published:** 2016

**Authors:** Gleim Dias de SOUZA, Luciana Rodrigues Queiroz SOUZA, Ronaldo Máfia CUENCA, Bárbara Stephane de Medeiros JERÔNIMO, Guilherme Medeiros de SOUZA, Vinícius Martins VILELA

**Affiliations:** 1 Base Hospital of Federal District, Brasília, DF, Brazil; 2 Catholic University of Brasília, Brasília, DF, Brazil

**Keywords:** Review Atlanta, Acute pancreatitis, Pancreatic abscess, Phlegmon, Peripancreatic collections, Necrotic collections, Pseudocysts, Walled necrosis.

## Abstract

**Introduction:**

Contrast computed tomography and magnetic resonance imaging are widely used due to its image quality and ability to study pancreatic and peripancreatic morphology. The understanding of the various subtypes of the disease and identification of possible complications requires a familiarity with the terminology, which allows effective communication between the different members of the multidisciplinary team.

**Aim:**

Demonstrate the terminology and parameters to identify the different classifications and findings of the disease based on the international consensus for acute pancreatitis ( Atlanta Classification 2012).

**Methods:**

Search and analysis of articles in the "CAPES Portal de Periódicos with headings "acute pancreatitis" and "Atlanta Review".

**Results:**

Were selected 23 articles containing radiological descriptions, management or statistical data related to pathology. Additional statistical data were obtained from Datasus and Population Census 2010. The radiological diagnostic criterion adopted was the Radiology American College system. The "acute pancreatitis - 2012 Rating: Review Atlanta classification and definitions for international consensus" tries to eliminate inconsistency and divergence from the determination of uniformity to the radiological findings, especially the terminology related to fluid collections. More broadly as "pancreatic abscess" and "phlegmon" went into disuse and the evolution of the collection of patient fluids can be described as "acute peripancreatic collections", "acute necrotic collections", "pseudocyst" and "necrosis pancreatic walled or isolated".

**Conclusion:**

Computed tomography and magnetic resonance represent the best techniques with sequential images available for diagnosis. Standardization of the terminology is critical and should improve the management of patients with multiple professionals care, risk stratification and adequate treatment.

## RESUMO

**Introdução:**

A tomografia computadorizada contrastada e a ressonância magnética são exames amplamente utilizados no estudo da morfologia pancreática e peripancreática. O entendimento dos diversos subtipos da doença e identificação de suas possíveis complicações requer familiaridade com a terminologia padrão, a qual permite comunicação efetiva entre os diversos membros da equipe multidisciplinar.

**Objetivo:**

Demonstrar terminologia e os parâmetros para identificação das diferentes classificações da doença a partir do consenso internacional para as pancreatites agudas (Classificação de Atlanta 2012.

**Método:**

Busca e análise de artigos no "Portal de Periódicos da CAPES" *com descritores "pancreatite aguda" e "Revisão de Atlanta".*

**Resultado:**

*: Foram selecionados 23 artigos que continham descrições radiológicas, manejo ou dados estatísticos relacionados à doença. Dados estatísticos adicionais foram obtidos no sistema Datasus e* Censo Demográfico 2010. O critério de diagnóstico radiológico adotado foi o do Colégio Americano de Radiologia. A "Classificação da pancreatite aguda - 2012: revisão da classificação de Atlanta e definições por consenso internacional" tenta eliminar a inconsistência e divergências a partir da determinação de uniformidade para os achados radiológicos, em especial à terminologia relacionada às coleções de fluidos. Termos mais abrangentes como "abscesso pancreático" e "flegmão" entraram em desuso e a evolução da coleção de fluidos pode ser descrita como: "coleções peripancreáticas agudas", "coleções necróticas agudas", "pseudocisto" e "necrose pancreática murada ou isolada".

**Conclusão:**

A tomografia computadorizada e a ressonância magnética representam as melhores técnicas com cortes sequenciais disponíveis para diagnóstico. A adequação da terminologia é ponto crítico e deve permitir o manejo do paciente por múltiplos profissionais, estratificação de risco e adequação de tratamento.

## INTRODUCTION 

Acute pancreatitis is defined as an inflammatory process of the pancreas and has broad spectrum of manifestations and clinical variations^30^. It is considered the most common pancreatic disease in children and adults^11^. The incidence ranges from 50 to 80 cases on a year per 100,000 population in the United States^12^. The incidence on Brazilian territory is geographically variable; however, according to Datasus and IBGE, the average of cases on a year per 100,000 population are of 19 (data referring to 2014)^6^ ^,^ ^7^. 

Half of all cases of acute pancreatitis among adults are related to biliary disease and alcoholism, while the pediatric service faces greater range of causes. Most important causes in children described by the availiable literature are (in order of frequency): biliary disease, medications, idiopathic, systemic diseases, trauma, metabolic disorders, hereditary and infectious causes^3^ ^,^ ^13^ ^,^ ^14^ ^,^ ^15^ ^,^ ^22^ ^,^ ^31^. 

The severe form, regardless of the cause, can reach 25-45% of morbidity and mortality. About 5-10% of these individuals develop necrosis and affect their pancreatic parenchyma in 5% of cases, peripancreatic tissue 20% of the cases and both of them in 70%^11^ ^,^ ^12^. 

Imaging tests have fundamental importance in diagnosis, determination of severity, recognition of complications and the therapeutic choice. They have a direct impact on clinically suspected cases and differential diagnosis^11^ ^,^ ^30^ ^,^ ^32^.

The aim of this study was to demonstrate the terminology and the parameters for identifying the different classifications of the disease from the International Consensus for Acute Pancreatitis (Atlanta Classification 2012

## METHODS

The methodology used on the paper was the search and analysis of articles in the "Portal de Periódicos da CAPES" with the headings: "acute pancreatitis" and "Atlanta Review".

## RESULTS

Were selected 23 articles containing radiological descriptions, management or statistical data related to the disease. Additional statistical data were obtained from Datasus and Population Census of 2010. The adopted criteria of radiological diagnostic were the ones recommended by the American College of Radiology.

### Diagnosis of acute pancreatitis

Diagnosis requires two of the three criteria: abdominal pain consistent with pancreatitis; serum lipase or amylase at least three times the normal limit; radiological findings on CT scans with contrast, MRI or transabdominal ultrasound^9^. Abdominal pain may be characteristic in late presentations, however amylase and serum lipase should probably be less than three times the normal range, thus the imaging test is needed to confirm the diagnosis^24^ ^,^ ^28^.

### Determining the severity of acute pancreatitis 

The classification defines three levels of severity for the disease: mild, moderate and severe. The categorization of these patients includes the presence of organic temporary failure (faults present for less than 48 h) or persistent (that persists for more than 48 h) and local (liquid or necrotic peripancreatic collections) or systemic complications (which may be related to pre-existing co-morbidities)^1^ ^,^ ^2^ ^,^ ^24^ ^,^ ^25^ ^,^ ^28^.

### Choice of imaging

The choice of imaging technique is dependent on the research reasons, clinical symptoms, duration of symptoms and laboratory findings^9^. 

Thus, its recommended perform the abdominal ultrasound for all patients with first presentation of acute pancreatitis, typical abdominal pain, increased pancreatic amylase and lipase, between 48-72 h of presentation and unknown cause^28^. The examination assesses the presence of calculi, biliary dilatation, gas presence and analysis of fluid collection^16^. 

The analysis of pancreatic morphology by the computed tomography imaging allows the diagnosis, determine the extent and severity of the disease^8^. However, it is not indicated on the mild presentations^11^ ^,^ ^28^. 

Clinical presentations that consist of more than 72 h of evolution, critical patients, carriers of critical clinical "scores", high severity index, signs of rapid deterioration, systemic inflammatory response syndrome and leukocytosis are the determinants of the precedence of computed tomography with contrast above other techniques^2^. Patients over 40 years in their first episode of acute pancreatitis without determined causes should perform the technique to exclude neoplastic causes^11^. 

MRI is the technic of choice for cases that there are limitations or contraindications to the application of computed tomography, need of multiple tests for monitoring disease progress and negative CT results with acute pancreatitis presentations ^2,9,28,32^. 

### Acute pancreatitis classification

**FIGURE 1:**
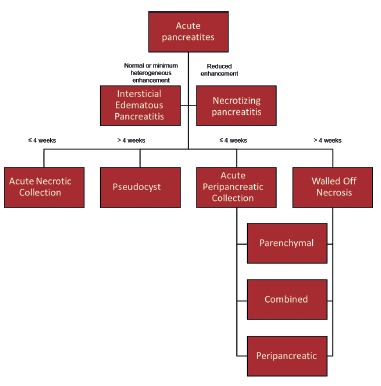
Pancreatitis classification and its associated collections

The Review of the Atlanta Classification, 2012, subdivides acute pancreatitis in two subtypes: edematous and necrotic. For both presentation the literature elucidates two stages that overlap and are closely related with two peaks of mortality: early and late. The initial phase usually ends at the end of the first week of symptoms onset, however it can reach the second phase with a resolution of pancreatic and peripancreatic ischemia, development of fluid collection or evolution for permanent necrosis and liquefaction^1^ ^,^ ^11^. The classifications and associated collections are described by Zhao et al.^32^ (Figure 1).

### Acute interstitial edematous pancreatitis

It is mild form of the disease that is usually resolved in the first week. Its main feature is the local or diffuse enlargement of the pancreas without the presence of necrosis. This increase is due to the intense inflammatory process causing interstitial or peripancreatic edema.

#### Features of computed tomography with contrast

**FIGURE 2:**
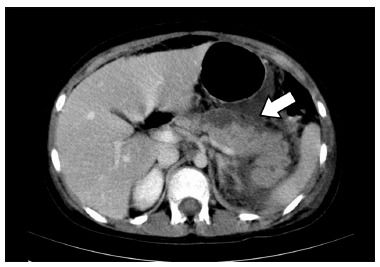
Interstitial edematous pancreatitis: CT exam in axial T2 cut

Generally it is characterized as enlarged pancreas with normal relative enhancement and regular peripancreatic fat, thickened or ground-glass opacity due to the inflammatory process. The amount of pancreatic fat can be variable but without enhancement^1^ ^,^ ^2^ ^,^ ^11^ ^,^ ^28^ ^,^ ^32^. The absence of necrotic tissue differentiates acute edematous pancreatitis from necrotic (Figure 2).

#### Characteristics of the magnetic resonance

The intensity of the pancreas at this stage is similar to normal organ. The "phase in" generally has enlarged pancreas and attenuated fat. Images in "phase in" with fat suppression have the delineation of the pancreas and its enhanced edges. In the pre- contrast phase, the body shows high signal intensity increases monotonically in the post-contrast image (Gadolinium) representing normal pattern of capillarity. image sequences in " phase out " are sensitive to the presence of edema or fluid collections^1^ ^,^ ^11^ ^,^ ^32^.

### Necrotizing pancreatitis 

Presentation with worse prognosis, characterized by inflammation with resultant necrosis of pancreatic or peripancreatic tissue. The damage to pancreatic perfusion and peripancreatic necrosis signs develop over several days, although the usage of early performed the contrasted CT may underestimate the severity of the disease^9^ ^,^ ^27^. 

Both computed tomography and magnetic resonance imaging are essential to obtain suitable images of the arterial phase, since the maximum highlight the pancreas is obtained in the late arterial phase and the largest signal difference between viable and necrotic tissue is evident in that stage^11^ ^,^ ^32^. 

#### Features of computed tomography with contrast

After contrast administration (Gadolinium) the findings include the highlight of parenchymal areas compromised. Changing the infusion and the formation of peripancreatic fluids collections can take several days to be evidenced by imaging.

#### Characteristics of the magnetic resonance

Necrosis can be identified as areas of hypointensity on "phase in" and increased signal intensity areas on "phase out", both associated with well defined areas of not enhanced parenchyma in postcontrast enhanced sequences (Gadolinium)^1^ ^,^ ^2^ ^,^ ^11^ ^,^ ^24^ ^,^ ^28^ ^,^ ^32^. 

### Pancreatic and peripancreatic collections

It is very important to differentiate fluid collections composed only of exudative fluid from those that have solid components from the necrosis process. The latest revision of Atlanta, 2012, uses the following terms for collections classification: "acute peripancreatic collections", "acute necrotic collections", "pseudocyst", "pancreatic necrosis walled or isolated" and "infected pancreatic necrosis."

### Acute peripancreatic collection 

Defined as a collection of fluid that develops during the initial phase of the disease, most cases after 48 h of onset of symptoms^4^. An important feature is the absence of solid component in the peripancreatic region^1^ ^,^ ^2^ ^,^ ^11^ ^,^ ^24^ ^,^ ^28^ ^,^ ^32^. Those collections are typically sterile and reabsorbed spontaneously after the treatment of acute pancreatitis^1^ ^,^ ^5^ ^,^ ^28^.

#### Features of computed tomography with contrast

Characterized as fluid collection, single or multiple, homogeneous and with low attenuation, without well-defined and confined in normal retroperitoneal fascial planes^18^.

#### Characteristics of the magnetic resonance

Images following "phase out" sequence are very sensitive to peripancreatic fluids, which are evidenced by an increase in signal intensity. The sequence "phase in" shows hypointense signal on a background and hyperintense fat^1^ ^,^ ^11^. 

### Pseudocyst

The term refers to fluid collections in peripancreatic region (which occasionally may be partially or entirely intrapancreatic) that persist for more than four weeks, form visible wall that imprisons the content and have no solid component^1^ ^,^ ^11^ ^,^ ^32^.

Most pseudocysts resolve spontaneously; however, bleeding and infections may complicate the condition of the patient. Infected pseudocysts may have gas in computed tomography^28^.

In case of suspicion and lack of characteristic clinical findings, it is necessary to perform a fine needle aspiration and morphological characterization of the content^1^ ^,^ ^11^ ^,^ ^28^.

#### Features of computed tomography with contrast

**FIGURE 3:**
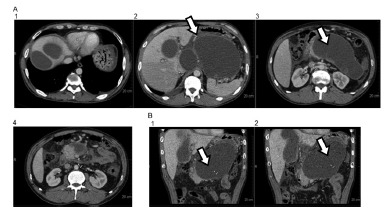
Pancreatic pseudocyst: tomographic analysis A1-4, T2 axial, B1-2 - sagittal section

There are homogeneous collections of low attenuation with uniform capsular enhancement. The increase in intensity is typically observed during the interstitial phase in detriment of the presence of granulation tissue (Figure 3).

#### Characteristics of the magnetic resonance

Sequential images in "phase in" show low signal intensity and in "phase out" usually have homogeneous increased signal intensity. The walls have minimal enhancement after contrast, due to the presence of fibrotic tissue^1^ ^,^ ^11^ ^,^ ^28^ ^,^ ^32^. 

### Acute necrotic collections

The term refers to collections containing liquid and necrotic tissue, which can be derived from the pancreatic parenchyma or adjacent tissue present in both intrapancreatic as peripancreatic region, and in most cases maintains communication with the pancreatic duct or its ramifications^1^ ^,^ ^2^ ^,^ ^11^ ^,^ ^24^ ^,^ ^28^ ^,^ ^32^. There is the possibility of rupture of the pancreatic duct and infection content.

Magnetic resonance imaging has much higher sensitivity in the detection of necrotic tissue when compared to computed tomography^5^ ^,^ ^9^ ^,^ ^11^.

#### Features of computed tomography with contrast

The main feature is the presence of heterogeneous attenuation, variable, higher than typical mitigations from only fluid collections. They may present as homogeneous attenuation without enhancement during the first week. The amount of solid content is variable and may be loculated^1^ ^,^ ^11^ ^,^ ^28^ ^,^ ^32^.

#### Characteristics of the magnetic resonance

**FIGURE 4:**
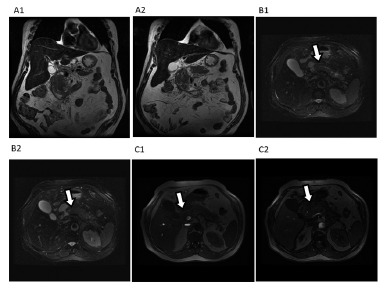
Acute necrotic collection: MRI examination A1-2 sagittal section in "phase out" B1-2: T2 Fat Sat, C1-2: T2 FSE axial

Necrotic debris are generally viewed as irregular regions of low intensity (Figure 4). Sequences "phase out" respiration independent, such as single catch, are useful for evaluating these collections once they are sensitive to the differentiation of solid content and in this stage of the disease the patient is generally weak and unable to control breathing adequately^5^ ^,^ ^9^.

### Pancreatic necrosis walled or isolated

Necrotic collections develop reactive and thick fibrotic wall that stores necrotic content inside after four weeks of evolution. The Atlanta Review uses the term "inflammatory wall" to describe this kind of collection. It has a higher incidence in the tail and body of the pancreas^1^ ^,^ ^9^ ^,^ ^28^ ^,^ ^32^. The treatment of such debris is more complex and depends on the surgical intervention^2^.

#### Features of computed tomography with contrast

It is presented as heterogeneous fluid and solid mitigations with different degrees of loculations with wall encapsulating well defined, which can extend to both pancreatic tissue as the extrapancreatic^1^ ^,^ ^11^ ^,^ ^28^ ^,^ ^32^.

#### Characteristics of the magnetic resonance

The sensitivity of magnetic resonance helps minimize diagnostic errors. Generally there are areas with heterogeneous intensity isolated by an intense accent wall in post-contrast, suggestive of isolation with solid and liquid content^1^ ^,^ ^28^ ^,^ ^32^.

### Infected pancreatic necrosis

The development of secondary infection in pancreatic necrosis is associated with high morbidity and mortality^9^ ^,^ ^27^ ^,^ ^28^. Thus the diagnosis in the early stages of infection is a determining factor in the conduct by antibiotic therapy^1^ ^,^ ^23^ ^,^ ^32^.

#### Features of computed tomography with contrast and magnetic resonance

The diagnosis of infection can be accomplished by gas visualization in both techniques. The extraluminal gas present in areas of necrosis might not form air-fluid levels depending on the stage of infection and the amount of necrotic tissue and fluid. In cases of doubt, confirmation can be obtained by fine-needle aspiration and microscopic analysis of the fluid or culture.

## CONCLUSION

Imaging tests are essential in the diagnosis and staging of acute pancreatitis. Computed tomography and magnetic resonance imaging are widely used, representing the best techniques with sequential cuts available for diagnosis. Tomography is the technique with greater acceptability and usage; however, MRI has the advantage in situations with CT contraindication and thorough soft tissue differentiation. The adequacy of terminology is critical and should facilitate the management of patients with multiple professionals, risk stratification and adequate treatment.
